# Adenomatous Polyposis Coli *(APC)* Is Required for Normal Development of Skin and Thymus

**DOI:** 10.1371/journal.pgen.0020146

**Published:** 2006-09-15

**Authors:** Mari Kuraguchi, Xiu-Ping Wang, Roderick T Bronson, Rebecca Rothenberg, Nana Yaw Ohene-Baah, Jennifer J Lund, Melanie Kucherlapati, Richard L Maas, Raju Kucherlapati

**Affiliations:** 1 Harvard-Partners Center for Genetics and Genomics, Harvard Medical School, Boston, Massachusetts, United States of America; 2 Division of Genetics, Department of Medicine, Brigham and Women's Hospital, Harvard Medical School, Boston, Massachusetts, United States of America; 3 Rodent Histopathology Core, Dana-Farber Harvard Cancer Center, Boston, Massachusetts, United States of America; Stanford University School of Medicine, United States of America

## Abstract

The tumor suppressor gene *Apc* (adenomatous polyposis coli) is a member of the Wnt signaling pathway that is involved in development and tumorigenesis. Heterozygous knockout mice for *Apc* have a tumor predisposition phenotype and homozygosity leads to embryonic lethality. To understand the role of Apc in development we generated a floxed allele. These mice were mated with a strain carrying Cre recombinase under the control of the human *Keratin 14 (K14)* promoter, which is active in basal cells of epidermis and other stratified epithelia. Mice homozygous for the floxed allele that also carry the *K14-cre* transgene were viable but had stunted growth and died before weaning. Histological and immunochemical examinations revealed that *K14-cre*–mediated *Apc* loss resulted in aberrant growth in many ectodermally derived squamous epithelia, including hair follicles, teeth, and oral and corneal epithelia. In addition, squamous metaplasia was observed in various epithelial-derived tissues, including the thymus. The aberrant growth of hair follicles and other appendages as well as the thymic abnormalities in *K14-cre; Apc^CKO/CKO^* mice suggest the *Apc* gene is crucial in embryonic cells to specify epithelial cell fates in organs that require epithelial–mesenchymal interactions for their development.

## Synopsis

Patients with familial adenomatous polyposis (FAP) and its variant, Gardner's syndrome, will develop hundreds of colorectal polyps. It is a heritable disease that is linked to a genetic mutation in the tumor suppressor gene *APC* (adenomatous polyposis coli). These individuals also develop extracolonic symptoms, among which are congenital hypertrophy of the retinal pigment epithelium, desmoid tumors, epidermoid cysts, disorders of the maxillary and skeletal bones, and dental abnormalities, suggesting the importance of APC functions in these organs. To understand the role of Apc in development and in organs other than intestine, we generated *Apc* mutant mice that can conditionally delete the gene when exposed to Cre recombinase. These mice were mated with *K14 (Keratin 14)*–*cre* mice that express Cre recombinase in skin and its appendages. The authors found that the mutant mice that have lost Apc in *K14-cre* transgene–expressing tissues were viable, but had stunted growth and died before weaning. These mutant mice showed developmental abnormalities not only in skin but also in many epithelial-derived tissues, including teeth and thymus. This work demonstrates the importance of Apc in development of many organs, and provides new insights into diagnosis and management of patients with *APC* mutations.

## Introduction

Adenomatous polyposis coli (APC) is a member of the Wnt signaling pathway and one of its known functions is to regulate the levels of β-catenin. Alterations in β-catenin regulation are very common in human tumors [1]. Loss of APC is associated with stabilization of the cytosolic β-catenin that ultimately results in its migration to the nucleus and activating a cascade of events leading to tumorigenesis. APC also interacts with a multitude of other cellular proteins, including axin-2 *(AXIN2),* plakoglobin *(JUP),* Asef *(ARHGEF4),* kinesin superfamily–associated protein 3 *(KIFAP3),* EB1 *(MAPRE1),* microtubules, and the human homolog of *Drosophila* discs large *(DLG1).* These interactions suggest that APC can potentially regulate many cellular functions, including intercellular adhesion, cytoskeletal organization, regulation of plakoglobin levels, regulation of the cell cycle and apoptosis, orientation of asymmetric stem cell division, and control of cell polarization [2,3].

*APC* is a tumor suppressor gene. Somatic mutations in *APC* are frequently found in many sporadic tumors of the colon and rectum. Autosomal dominant germline mutations in *APC* cause familial adenomatous polypois (FAP) and its variant, Gardner syndrome. FAP patients are characterized by hundreds of adenomatous colorectal polyps, with an almost inevitable progression to colorectal cancer in the third and fourth decades of life [4,5]. In addition to colorectal neoplams, these individuals can develop extracolonic symptoms, among which are upper gastrointestinal tract polyps, congenital hypertrophy of the retinal pigment epithelium, desmoid tumors, disorders of the maxillary and skeletal bones, and dental abnormalities [6], suggesting the importance of *APC* gene functions in these organ systems.

Although the role of APC in the initiation of human colorectal cancer is well established, its role in other tissue and developmental processes are not well understood. Given the importance of regulation of Wnt signaling in embryonic pattern formation and morphogenesis of many organs, mechanistic understanding of APC in development and in extracolonic tissues becomes critical to better assess potential adverse events in humans. One approach to understand the role of *Apc* in development is to develop mice with an inactivating *Apc* mutation. Several genetically modified mouse strains for *Apc* have been described [7–10]. Most of these models, in the heterozygous state, show a gastrointestinal and other tumor predisposition phenotype [7–10]. Mouse embryos that are homozygous for the genetic modification die during embryogenesis, and some of the models do not survive beyond gastrulation [8,11]. An alternate approach to understand the role of Apc in development and/or in specific tissues is to generate a mouse strain that carries a conditionally modified allele and mate it with a mouse strain that facilitates the modification of the conditional allele in specific cell lineages.

To assess the role of Apc in different stages of life systematically, we generated mice containing a conditional knockout (CKO) mutant allele of *Apc (Apc^CKO^).* These mice were mated with a strain carrying Cre recombinase under the control of the human *Keratin 14 (K14)* promoter, which is active in basal cells of epidermis and other stratified epithelia. We report here that *K14* promoter-driven loss of *Apc* resulted in aberrant development of several organs that require inductive epithelial–mesenchymal interactions, including hair follicle, teeth, and thymus, and resulted in neonatal death in mice. We found that Apc plays a crucial role in determinations of cell fates during the embryonic development, possibly via temporal and tissue-specific regulation of β-catenin levels in the skin, its appendages, and in the thymus.

## Results

### Generation of the *Apc^CKO^* and *Apc^Δ580^* Mice

**Figure 1:**
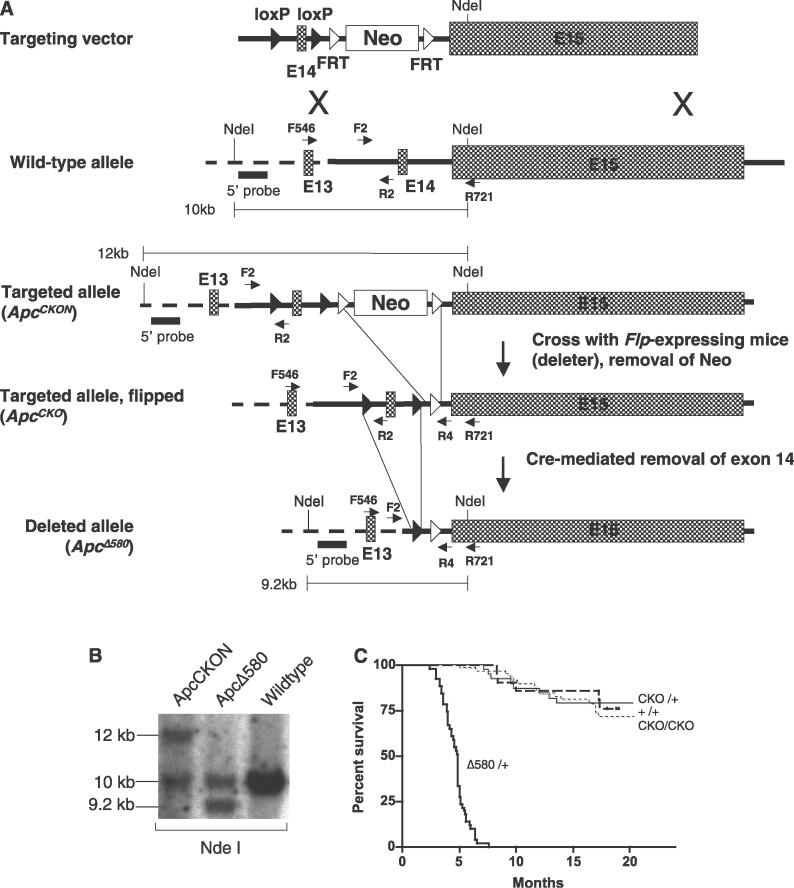
Generation of the Conditional *Apc* Allele (A) Schematic diagram of exons 14 and 15 of the mouse *Apc* gene, the targeting vector, and the resulting conditional allele with 2 LoxP sites sandwiching the exon 14. The PGK-neomycin cassette was inserted within intron 14 by recombineering technique. This cassette is sandwiched by 2 FRT sites that could be removed by crossing to FLPe-expressing mice. Positions of PCR primers used for genotyping PCR (F2, R2, R4) and RT-PCR (F546 and R721) are indicated. Positions of probe used for Southern blot analysis with NdeI sites are also shown. Upon Cre-mediated recombination, exon 14 is removed and leads to truncated Apc protein, of which the first 580 aa correspond to the normal. (B) Southern blot analysis of NdeI-digested genomic tail DNA isolated from F1 mice of various *Apc* mouse lines *(Apc^CKON^, Apc^Δ580^),* hybridized to a 600-bp probe. Tail genomic DNA from *Apc^CKON^* F1 mice derived from a modified ES clone showed a 12-kb band for the *Apc^CKON^* allele and a 10-kb band for the wild-type allele, whereas genomic DNA from the *Apc^Δ580^* mouse was heterozygous for the *Apc^Δ580^* allele (9.2-kb band). (C) Kaplan-Meier survival plot of *Apc^CKO/+^* mice (thin solid line, *n* = 39), *Apc^CKO/CKO^* mice (thin dotted line, *n* = 57), *Apc^Δ580/+^* mice (solid line, *n* = 51), and wild-type littermates (broken line, *n* = 21). Heterozygosity of the *Apc^Δ580^* allele led to a significantly shortened survival (*p* < 0.0001), whereas those of heterozygous and homozygous *Apc^CKO^* mice had no significant difference to that of wild-type littermates.

To investigate the role of Apc in development of skin and its appendages, we used the Cre/loxP technology to introduce a conditional mutation of the *Apc* gene in mice. We constructed embryonic stem (ES) cells and mice carrying an *Apc* allele harboring both a pair of loxP sites flanking *Apc* exon 14 and a pair of FLP recognition target (FRT) sites flanking PGK-neomycin selection cassette by recombineering [12,13] (Figure 1A, *Apc^CKON^* allele, N for neomycin cassette). A PGK-neomycin cassette was inserted in the same transcriptional orientation as *Apc* in intron 14 of the endogenous gene. The *loxP* and *FRT* sites were used to aid unidirectional recombination [12,13]. Two mouse lines containing the same modification were generated from two independent ES clones to ensure that these two lines behave in the same way. These *Apc^CKON/+^* mice were crossed with FLPe-deleter to generate *Apc^CKO/+^* mice that were heterozygous for the final *Apc* conditional *(Apc^CKO^)* allele that removed the PGK-neomycin cassette and contains only the loxP sites in the introns flanking exon 14. To assess the effect of deleting exon 14 in mice, both lines of *Apc^CKO/+^* mice were crossed with the Cre-deleter to generate the germline knockout line of *Apc,* designated *Apc^Δ580/+^.* The mutant allele *(Apc^Δ580^)* lacks exon 14 (Figure 1A). The transcript from loss of exon 14 results in a shift in the normal reading frame, resulting in a premature chain termination codon which, if utilized, would result in a truncated polypeptide that is 605 aa in length, of which the first 580 aa correspond to the normal Apc protein.

Southern blot analysis of tail DNA from F1 offspring of both *Apc^CKON^* and *Apc^Δ580^* lines confirmed the germline transmission of modified *Apc* allele (Figure 1B). Mice that are heterozygous for *Apc^Δ580^* mutation are viable but have a significantly reduced lifespan (Figure 1C). These results suggested that deletion of exon 14 indeed results in either loss or abnormal function of the *Apc* gene product. *Apc^Δ580/+^* mice have median survival of 5 mo of age (Figure 1C), with progressive signs of rectal bleeding and anemia. Similar to the results reported with an independently generated *Apc^Δ580/+^* conditional mouse strain [14], *Apc^Δ580/+^* mice had more than 100 (120 ± 37, *n* = 11) intestinal tumors at the time of their death (Figure S1). Inactivation of wild-type *Apc* is an important prerequisite for tumor development. We analyzed 30 intestinal tumors from *Apc^Δ580/+^* mice by in vitro transcription and translation assay, but none of them showed truncated Apc products (unpublished data), indicating that the most likely mechanism of wild-type *Apc* inactivation is by allelic loss. The mutant allele had to be maintained and transmitted through male mice, as *Apc^Δ580/+^* females were frequently not healthy enough to successfully nurse their own pups because of their tumor burden.

*Apc^CKO/+^* mice were intercrossed to generate *Apc^CKO/CKO^* offspring. Approximately one-quarter of the offspring (17 of 81) were homozygous for the *Apc^CKO^* allele. These mice as well as heterozygous mice for *Apc^CKO^* allele are normal, showing no differences in their survival to the wild-type littermates (Figure 1C). We tested whether our *Apc^CKO^* allele can compliment the wild-type allele by crossing the *Apc^CKO/CKO^* female with *Apc^Δ580/+^* male mouse. The resultant *Apc^CKO/Δ580^* offspring were viable and born in the Mendelian ratio, suggesting that the presence of loxP sites in introns flanking exon 14 have no adverse effect on the function of the *Apc* gene.

### *K14*-Driven Loss of *Apc* Results in Severe Growth Retardation and Early Lethality

To introduce the mutation of *Apc* into cells expressing K14, we crossed WW6 ES cell–derived [15] *Apc^CKO/+^* mice with *K14-cre* recombinase mice in FVB background [16]. The *K14-cre; Apc^CKO/+^* mice were normal in appearance and were fertile. *K14-cre; Apc^CKO/+^* males were crossed to *Apc^CKO/CKO^* females to avoid the potential deleter effect in oocytes of *K14*-cre–positive females [17]. The mice were intercrossed thereafter for maintenance; hence, the mice used for analysis were in a mixed background of FVB, 129/S, and C57BL/6 in similar proportions, with minimal contribution of SJ.

The K14 promoter is a commonly used epidermal cell promoter because of its expression by the mitotically active cells of the epidermis and its appendages in mature skin [18], but most notably it is active in embryonic ectoderm as early as the single layered ectodermal stage of embryonic day (E) 9.5 [19]. A restricted expression of K14 is also found in thymic epithelial cells (TECs) in the medulla of normal thymus [20].

**Figure 2:**
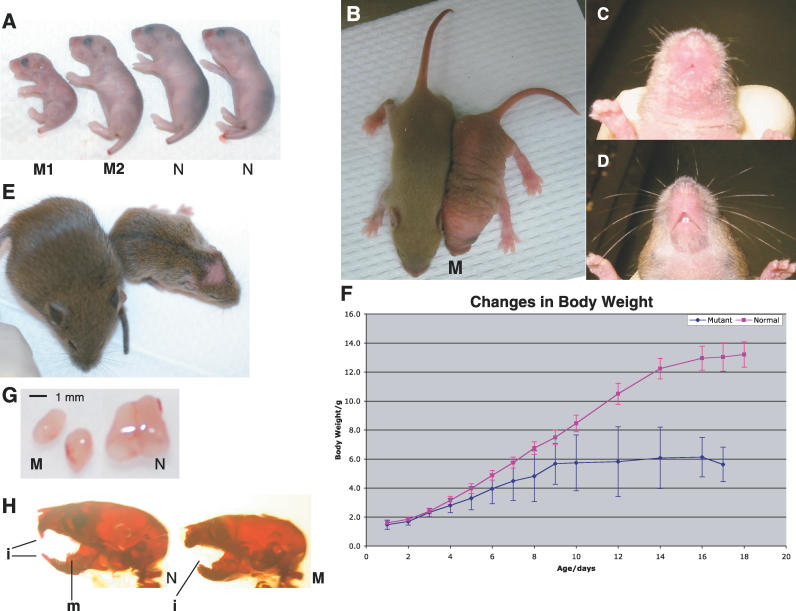
Postnatal Mortality and Stunted Growth in *K14-cre; Apc^CKO/CKO^* Mutant Mice Animals whose genotype is either heterozygous or homozygous for the wild-type *Apc* allele are referred to as normal (N); those whose genotype are *K14-cre; Apc^CKO/CKO^* and show the presence of *K14-cre*–recombined mutant *Apc* allele are called mutant (M). (A) Two P3 mutant mice, M1 and M2, and their normal littermates, showing size variation among mutants. (B) P8 mutant mouse (right) and a normal littermate. Note sparseness of hair coat and abnormal ears. (C–D) Vibrissae of whisker pads are short and oddly angled in a P12 mutant mouse (C), relative to control (D). Note the lack of incisors in the mutant. (E) A P17 mutant mouse (right) with its littermate. Its bare forehead, dorsal median line, and abnormal ears are evident. (F) Growth curve of mutants and normal littermates. Mutants exhibit stunted growth, which became more prominent as they aged, and weigh significantly less than littermates from P8 (*p* < 0.05). (G) Comparison of mutant and normal thymus from P3 mice. The mutant thymus (left) is dramatically smaller for its age compared to the normal littermate (right). The scale bar equals 1 mm. (H) Skeletal preparations of normal (left) and mutant (right), showing differences in development of both incisor (I) and molar (M) teeth.

We genotyped a total of 458 pups (8–10 d old) from 67 litters resulting from crosses between *K14-cre; Apc^CKO/+^* and *Apc^CKO/CKO^* mice. The mutant mice of the genotype *K14-cre; Apc^CKO/CKO^* (hereafter, KA mutant) were born, but the observed frequency of KA mutants was much less than expected (78 of 458 [17.0%]; *p* < 0.0005 Chi-square analysis, Table 1). To assess the basis for the neonatal lethality of KA mutants, we monitored three litters from birth to weaning by measuring the body weight of each pup every day. A total of 25 pups were born from three litters, of which 7 (28%) were confirmed to be *K14-cre; Apc^CKO/CKO^* by genotyping, indicating that KA mutants were born in the expected Mendelian ratio. The KA mutant pups were nursed normally, and there was milk in their stomachs during the first 2 or 3 d after birth, but they failed to thrive (Figure 2). By postnatal day (P) 8–10, at the time of genotyping, many KA mutant pups were considerably smaller than their littermates (Figure 2B–2F) and some have died at or prior to this age. None of KA mutants survived to weaning age.

**Table 1:**
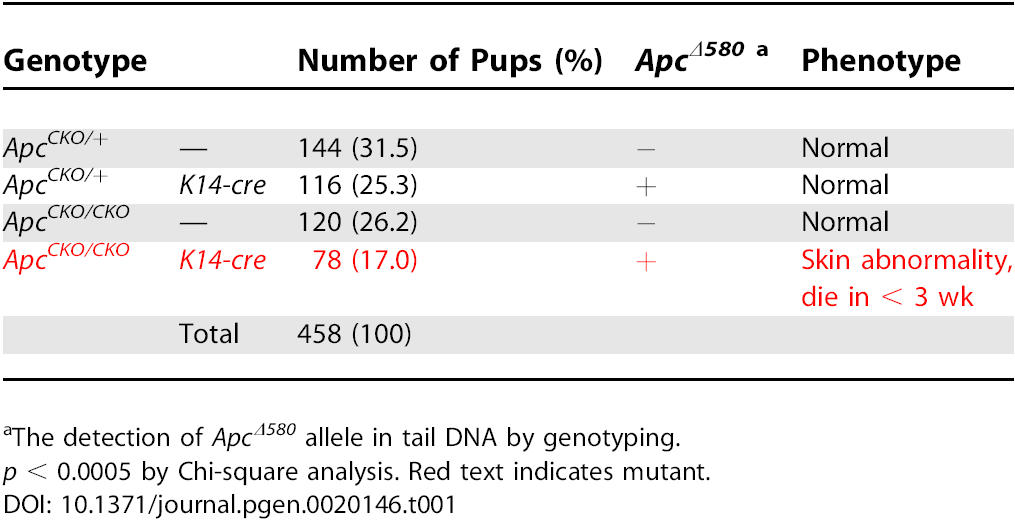
Genotype Distribution of Progeny from the Matings

The ability of whole embryos to exclude blue dye was used to examine the epidermal barrier, normally acquired beginning at E16 and complete by E18.5 [21]. Analyses of E17.5–E18.5 KA mutants showed that they were able to exclude blue dye, indicating that the epidermal barrier was intact (Figure S2). At these embryonic ages, there were no differences in size between the mutants and their littermates, but the mutants showed a patch of “birthmark” or dark pigmentation on their foreheads and a dark median line that ran caudally from head to tail. Their external ears or pinnae were shriveled in appearance and pigmented compared to those of littermates.

External characteristics of KA mutants that were evident at E18.5 persisted after birth and became more prominent as they grew (Figure 2A–2F). Growth of pelage hair was generally delayed in the mutants. At around P8, the KA mutants were hairless and had wrinkled skin while their phenotypically normal littermates had a smooth thin coat of hair (Figure 2B). At this age, two lower incisors start to erupt in normal littermates and these were absent in the KA mutants (Figure 2C and 2D). Animals also tended to be smaller and around P10–P12 displayed abnormally short and misshapen vibrissae and short, shaggy pelage hairs (Figure 2C and 2D). Development of thick ridges in their skin, particularly around the ears, eyelids, forehead, nose, and paws, became noticeable (Figure 2E). These regions looked scaly, and these animals hardly kept their eyes open. In contrast to the normal littermates that consistently increased their body weight with age, surviving KA mutants started to lose weight from P10 onwards; by P16–P17 they were all lethargic, and none of them survived to weaning (Figure 2E and 2F). At the time of autopsy all the mutants were toothless, without incisors or molars, and their stomachs were consistently small and had no solid food, unlike their age-matched littermates, suggesting that the observed weight loss could be the result of failure to ingest solid food (Figure 2F). Interestingly, changes in body weights and timing of hair growth varied considerably among mutant pups even if they were from the same litter, whereas those of phenotypically normal littermates tended to be similar. This difference was also reflected in the variation in timing of death in mutants: some mutant pups were born alive but died within a day or two, some survived close to the weaning age. This variability of the mutant phenotypes suggests possible variation in the timing and efficiency of *cre*-mediated *Apc* deletion. It is possible that the genetic background has a role to play in this variability.

Gross examination of internal organs also showed that the mutants' thymi were consistently inconspicuous and were very small for their age, whereas those of their littermates were very prominent in size (Figure 2G). This difference was evident as early as P3. Quite frequently mutant thymi in P12–P17 mutant mice also contained black deposits within the tissue (unpublished data). Mutant mice were also examined for any skeletal abnormalities by preparing skeletal specimens of P16–P17 mice stained with Alizarin red. No differences between the normal and KA mutant mice in the mandibular bone can be detected, but the mutant mice lacked or had underdeveloped set of maxillary incisors and molars (Figure 2H). We detected no other major skeletal abnormalities.

### Genotype- and Tissue-Specific Expression of the Truncated *Apc* Transcripts

**Figure 3:**
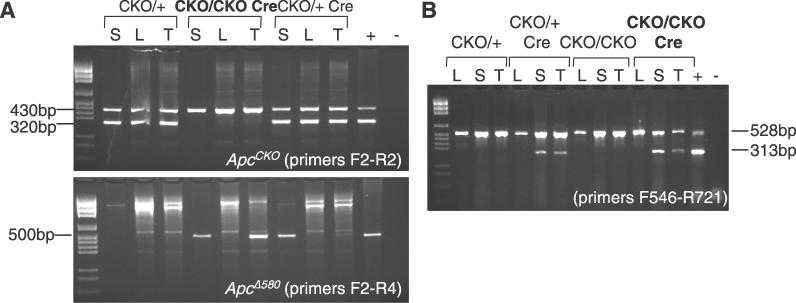
Tissue-Specific Detection and Expression of Deleted *Apc* Alleles (A) Tissue-specific genotyping PCR. Only genomic DNA samples from the skin (S) and thymus (T), but not liver (L) of mice positive for *K14-cre* show the presence of deleted *Apc^Δ580^* allele. **(**B**)** Genotype- and tissue-specific expression of the truncated *Apc* transcripts. A representative gel of RT-PCR using primers F546 and R721, showing that only RNA from the skin and thymus but not liver of mice positive for *K14-cre* have transcripts from both wild-type (528 bp) and deleted (313 bp) *Apc* alleles.

To assess the molecular effects of the *K14-cre*–mediated recombination, we screened for the presence of deleted *Apc (Apc^Δ580^)* alleles. Genomic DNA was extracted from liver, thymus, and skin from all 4 possible genotypes: *K14-cre; Apc^CKO/CKO^, K14-cre; Apc^CKO/+^, Apc^CKO/CKO^,* and *Apc^CKO/+^*. Genotyping on genomic DNA from these tissues showed that the *Apc^Δ580^* allele (500-bp product) was detected only from the skin and thymus of the *K14-cre*–positive mice. The presence of mutant *Apc* allele in the thymus of *K14-cre; Apc^CKO/+^* mice was consistently much less than the DNA from the skin of the same animal or other tissues from the KA mutants. In addition, this product was not detected at all in either the liver of *K14-cre*–positive or in any of the *K14-cre*–negative mouse tissues samples, establishing that Cre-mediated recombination has taken place in the tissue-specific manner in the mice that inherited *K14-cre* (Figure 3A).

*Apc* transcripts were also analyzed by RT-PCR with primers spanning exon 14 (Figure 1A) using total RNA isolated from the corresponding tissue samples. We detected the expected RT-PCR product (313 bp) from the truncated *Apc (Apc^Δ580^)* allele only in the tissues where Cre recombinase is known to be expressed in the *K14-cre*–positive mice. However, this product was not detected in either the *K14-cre*–negative mouse tissues samples or the liver of *K14-cre*–positive mice, and only the product from the wild-type allele (528 bp) was detected from these RNA samples, further confirming that Cre-mediated recombination has taken place in the tissue- and genotype-specific manner (Figure 3B).

### *K14-cre*–Driven *Apc* Loss Induced Aberrant Hair Follicles throughout the Epidermis

**Figure 4:**
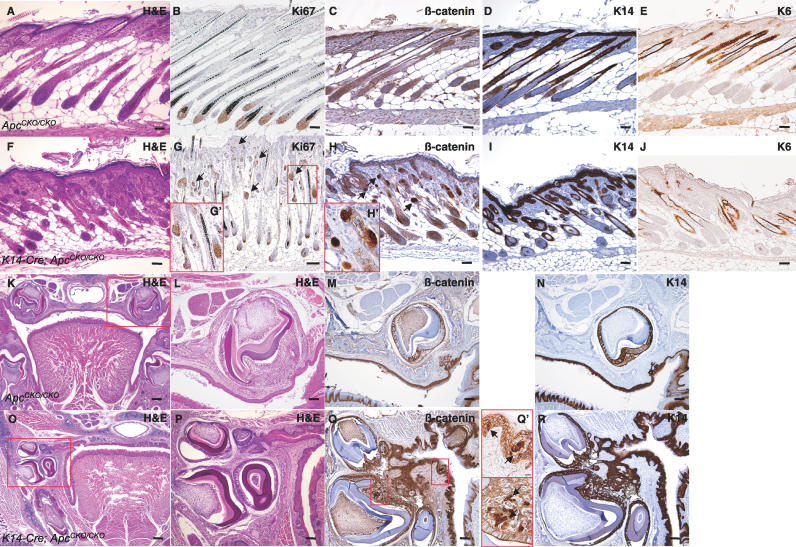
Histological and Immunochemical Examination of P12 Skin and Teeth (A–E) P12 normal skin. (F–J) P12 mutant skin. (K–N) P12 normal oral cavity. (O–R) P12 mutant oral cavity. Stained with H&E for histology (A, F, K–L, O–P), Ki67 (B, G), β-catenin (C, H, M, Q), K14 (D, I, N, R), and K6 (E, J). Aberrant follicular morphogenesis, characterized by formation of irregularly spaced, nonpolarized hair follicles, in mutant skin is evident. Despite the abnormal histology, proliferation seems to be confined to hair bulb-like structures (arrows in [G], inset [G′] at higher magnification), but in mutant skin (arrows in [H], inset [H′] at higher magnification) and oral cavity (arrows in insets [Q′] at higher magnification) elevated cytosolic localization of β-catenin is detected in some cells. Scale bars: 50 μm for (A–F), (H–J); 250 μm for (K) and (O); 100 μm for (G), (L–N), (P–R); 20 μm for (Q′).

To understand the basis for delayed and abnormal hair development in the KA mutants, we conducted a histological and immunohistochemical examination (Figure 4). The hair follicle is an epidermal appendage that consists of an upper permanent portion, and a lower cycling portion that produces the hair [22,23]. The outer root sheath (ORS) is contiguous with and biochemically similar to the basal layer of the epidermis. The inner layers of the hair follicle include three concentric layers of inner root sheath and three concentric layers of hair-producing cells. At the base of the hair follicle is the germinative hair follicle bulb, which contains rapidly proliferating “matrix” cells that differentiate to populate all of the layers of the inner root sheath and the hair shaft itself [22]. During the anagen phase of the hair cycle (until P15), hair follicles of phenotypically normal mice grew deeply into the subcutaneous fat and were uniformly spaced and aligned in parallel arrays at a specific angle relative to the skin surface (Figure 4A). In contrast, KA mutant follicles were irregularly spaced and often seen as disoriented and clamped invaginations at P3 that became even more remarkable at P12 when the mutant mice were covered by fur coat (Figure 4F). Bulbs were often bent in addition to being irregularly angled to one another and their sizes and locations were often variable. Clusters of multiple invaginations or dysplastic follicular structures were frequently observed throughout the epidermis, whereas other regions showed gaps with no follicles. Serial sectioning indicated that some of the hair follicles in the P12 mutant skin were not properly formed or shorter than normal. Taken together, these features could account for the apparently delayed, followed by outgrowth, of the short and shaggy-looking fur coat of these mutant mice.

Apc is a regulator of β-catenin that is important for Wnt signaling. We examined the patterns of expression of β-catenin in the affected tissues. In the normal skin, β-catenin, a member of the adherens junction complex, was found in the ORS of hair follicles and basal layer of epidermis, where K14 expression is also observed (Figure 4C and 4D), whereas the expression of K1, involucrin, and loricrin (markers for spinous and granular layers of epidermis) was only observed in the nonbasal epidermis (unpublished data). The patterns of expression of K14, K1, involucrin, and loricrin, in skin from mutant and normal littermate mice at P3–P17, showed no significant differences in the terminal differentiation (Figure 4A–4D, 4F–4I). Similarly, the pattern of expression of K6, which is normally only expressed in the suprabasal or inner layer of the ORS of the hair follicle but not in the epidermis (Figure 4E), did not change. Due to the abnormal and disorganized structure of hair follicles themselves, K6 localization highlighted the histological abnormality (Figure 4J). Yet as in the normal skin, K6 was principally seen only in the suprabasal layer of the ORS that did not colocalize with the basal markers, K14 or K5 (Figure 4I and 4J).

In normal skin, proliferating cells were detected in either the basal layer of epidermis or in germinative hair follicle bulbs at the base (Figure 4B). In the mutant skin, either BrdU incorporation or Ki67 expression was observed not only in cells in bulbs at the base of the hair follicle but also in bulb-like structures that were budding out from the ORS of the existing hair follicles (Figure 4G and 4G′). Each budding tip was becoming like a hair follicle bulb containing proliferating cells. Hence, despite the abnormal histology in the mutant skin, proliferation seems to be confined to bulb-like structures as in the normal skin (Figure 4G and 4G′). The exact locations of hair follicle bulbs were not as easy to define for some mutant follicles due to their disorganized structures. Interestingly, in the mutant skin, in addition to diffuse membrane-bound localization as in the normal skin, cells with strong cytosolic β-catenin localization were also observed frequently (Figure 4H and 4H′). These elevated β-catenin–expressing cells were usually surrounded by proliferating cells, forming bulb-like structures. Comparison of immunochemically stained serial sections showed that these intense cytosolic β-catenin stainings were usually found in either K14-positive K1-negative basal epidermis or K14-positive K6-negative basal ORS cells, and are surrounded by proliferating cells.

**Figure 5:**
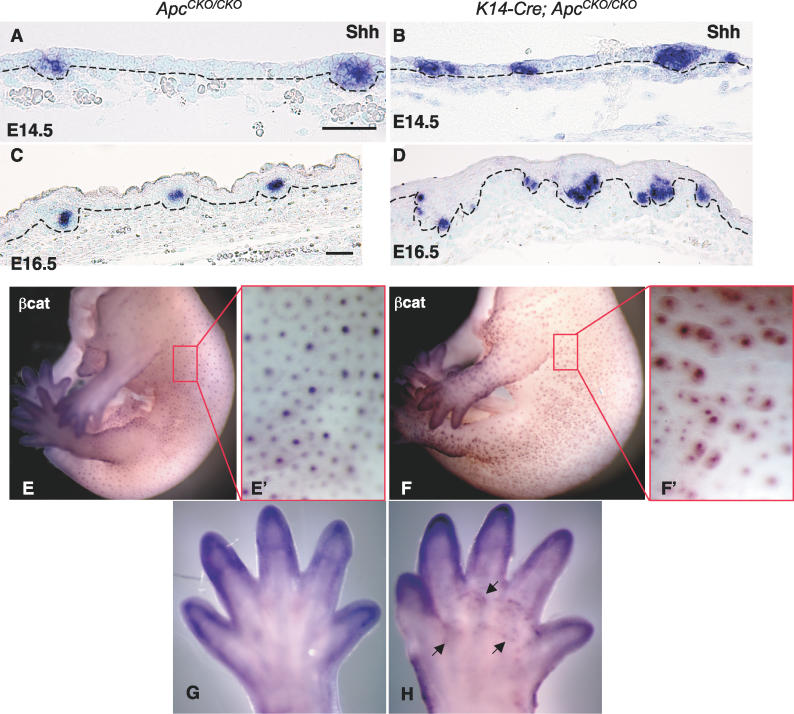
Expression of *Shh* and β-catenin Transcripts in Normal *(Apc^CKO/CKO^)* and Mutant *(K14-cre; Apc^CKO/CKO^)* Embryonic Skin (A–D) Section ISH with *Shh* probe in E14.5 normal (A), E14.5 mutant (B), E16.5 normal (C), and E16.5 mutant (D) skin. Broken lines indicate the interface between epithelium and mesenchyme. Scale bars: 50 μm. Whole mount in situ detection of β-catenin in E15.5 normal (E, G), mutant (F, H) embryos. Aberrant initiation of multiple hair placodes is evident at E14.5. Loss of *K14*-driven *Apc* loss caused aberrant pattern formation (F′) and formed ectopic hair placodes in normally hairless foot pads (H, arrows) which are absent in normal (G).

To determine the initiation of hair follicle morphogenesis in these mutants, we examined the expression pattern of *Sonic hedgehog (Shh),* a factor expressed in hair bulbs in embryonic skin (Figure 5). The aberrant hair follicle morphogenesis is evident as early as E14.5 in mutant embryonic skin, by multiple apolarized expression of *Shh* throughout the epidermis (Figure 5B), whereas that of control embryos was well polarized and regularly spaced (Figure 5A). With development, control mouse hair follicles invaginate downward in a polarized manner (Figure 5C), whereas those of mutant embryos were completely irregular and apolarized (Figure 5D). It was also noted that the size of each “budding” follicle, as detected by *Shh* expression, was variable (Figure 5D). The intensity of *Shh* staining was generally stronger in mutant skin than in the normal skin. The aberrant initiation of multiple hair placodes during early hair follicle morphogenesis was also evident by the whole-mount in situ hybridization (ISH) of E15.5 mutant embryos for β-catenin (Figure 5F and 5F′). The expression pattern of β-catenin in embryos clearly demonstrated the formation of regular arrays of hair placodes in the normal embryonic skin (Figure 5E and 5E′), but such regular patterning was lost, and often tightly clustered abnormal hair placodes were initiated in mutant embryonic skin (Figure 5F′). Aberrant hair placodes were also evident throughout the skin surface of limbs in E15.5 mutants (Figure 5F), whereas those of the control embryos had not yet formed (Figure 5E). Most interestingly, in the mutant footpads, where hair placodes do not normally form (Figure 5G), we also found ectopic irregularly sized and spaced hair placodes, indicating that the footpads still have the potential to form hair placodes in the absence of the *Apc* gene (Figure 5H).

These results collectively suggest that the terminal differentiation does take place normally in the mutant skin, but initiation of embryonic hair follicle morphogenesis is severely disrupted, accompanied by a continuous ectopic hair follicle morphogenesis in postnatal mutant skin.

### Effects of *K14-cre*–Driven Loss of *Apc* in Other Epidermal Appendages

Similar to the biology of hair follicles, *K14-cre*–driven loss of *Apc* also affected the development of other epidermal appendages that depend on epithelial–mesenchymal interactions for their formation. The most striking of these was dental dysplasia (Figure 4K–4R). Tooth development is normally initiated between E11 and E12 by invagination of ectodermally derived oral epithelium into the underlying cranial neural crest–derived mesenchyme, generating a tooth germ. Despite the grossly toothless phenotype of KA mutants, histological analysis of their oral cavities revealed the formation of multiple tooth buds at each location. These aberrant teeth obviously failed to grow out during the dietary transition from milk to solid food. Analogous to the expression patterns of K14 and β-catenin in the normal skin, diffuse membrane-bound expression of β-catenin was detected in K14-expressing oral epithelium and ameloblasts of normal mice. In mutants, some of the K14-expressing cells also showed strong cytosolic/nuclear β-catenin staining, as observed in the mutant skin (Figure 4Q, 4Q′, and 4R). Initiation of ectopic tooth buds in the mutant mice was evident at E15.5 by extra dots of *Shh* expression adjacent to the primary teeth (data not shown).

Loss of *Apc* also leads to hyperplasia in squamous epithelia of cornea, oral, salivary, and Hardarian glands (unpublished data). Squamous metaplasia to hair follicle–like structures, ectopic hair follicle morphogenesis was also observed in these epithelia.

### *K14-cre*–Driven *Apc* Loss Results in Hypoplastic/Athymic Mice

Thymus is an organ that is also known to have K14 expression [16]. It represents the primary lymphoid organ for thymocyte development and selection. Distinct population of TECs of cortex and medulla mediates both of these critical functions. Cortical and medullary TEC subsets are characterized by differential expression of four keratin species: K8, K18, K5, and K14.

**Figure 6:**
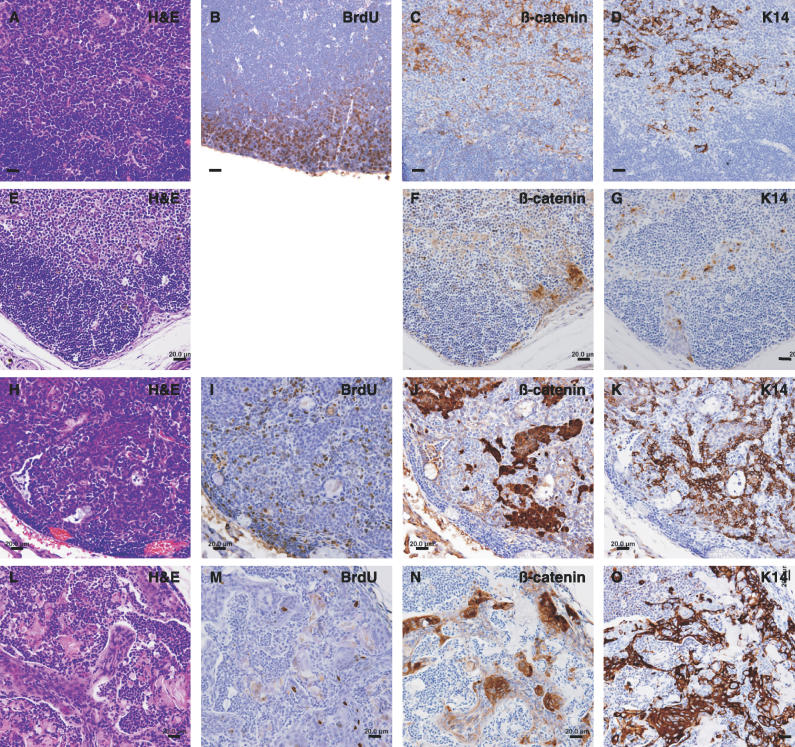
Histological and Immunochemical Examination of Thymus (A–D) P3 normal thymus. (E–G) Mild P3 mutant thymus. (H–K) Severe P3 mutant thymus. (L–O) P13 mutant thymus. Stained with H&E for histology (A, E, H, L), BrdU (B, I, M), β-catenin (C, F, J, N), and K14 (D, G, K, O). (B) Actively dividing thymocytes are visible at the superficial edge of cortex of normal P3 thymus. Note the progression of histological abnormalities in the mutant thymus from mild P3, severe P3 to P13 (A, E, H, L). Scale bars, 20 μm.

The normal thymus is a lobulated lymphoid organ, each lobule clearly showing the two distinct TEC compartments, an outer cortex and an inner medulla (Figure 6). There were no major differences in the histology of thymus between the ages P3 to P17 in phenotypically normal littermates. As shown in the H&E staining of thymus, the cortex was formed of dense lymphoid tissue that lacks nodules (Figure 6A). Since the stroma of the medulla is less heavily infiltrated with lymphocytes than the cortex, the medulla stained more lightly than the cortex. In normal mice, the thymus retains its size until the young adult age and regresses thereafter by atrophy. In the normal young mice we examined (P3–P17), it is evident that thymocytes were mitotically active in the cortex as determined by BrdU immunostaining (Figure 6B). Immunohistochemistry of normal thymus from P3 to P17 mice showed a similar staining pattern for K14 in that its expression was restricted to a small population of TECs in the inner medullary region and in the keratinocytes in Hassall's corpuscles (Figure 6D). Diffuse cytoplasmic staining for β-catenin was also detected in the medullary epithelial cells (Figure 6C). In contrast to K14 expression, diffuse staining for K8 was observed in epithelial cells both in the medulla and cortex (Figure S3). K1 staining was not detected in young mice at P3 but in older mice it was detected in differentiated keratinocytes in some of Hassall's corpuscles (Figure S3).

The histological abnormalities of thymus were evident as early as P3 in KA mutants (Figure 6E and 6H). The thymus was made of two lobules as in the normal mice but the mutant thymus was significantly smaller in size than that of the age-matched controls (Figure 2G). Interestingly, variations in the phenotypic severity of the mutant pups at P3 were prominently reflected in the extent of histological abnormalities of thymus. A P3 KA mutant pup that showed milder phenotype with a comparable body weight to its normal littermates (Figure 2A, M2) showed milder thymus abnormalities (Figure 6E) compared to its more severe mutant littermate (Figure 2A, M1; Figure 6H). The milder P3 mutant thymus was already much smaller in size compared to those of normal littermates (data not shown) but two epithelial compartments of thymus were histologically still distinguishable, with colonization of thymocytes evident in the cortex. However, there were small populations of lightly stained cells by H&E extending from the edge of the outer cortex towards inner medulla (Figure 6E), and these cells showed intense nuclear β-catenin staining whereas the rest of the medullary cells showed diffuse β-catenin staining pattern similar to that of the control (Figure 6F). Localization of K14 was limited to a few cells in the medulla and some overlapped with K8 localization (Figure 6G).

In the other P3 mutant thymus the distinct thymic epithelial compartments have been lost completely, and only a few lymphocytes were remaining at the edges and some in the middle (Figure 6H). Proliferative activities were no longer observed in thymocytes as prominently as in the normal thymus, but the epithelial cells seemed to be forming concentric structures (Figure 6I). Unlike in the normal or mild mutant thymi, the severe P3 mutant thymus showed extensive K14 expression that overlapped with K8 expression (Figure 6K). These cells were more like basal cells of the skin than TECs and were adjacent to the most immature looking cells that were showing strong nuclear and cytoplasmic β-catenin staining (Figure 6J). The nuclear staining of β-catenin was not observed in the normal age-matched thymus (Figure 6C). Most notably, the K14 and β-catenin staining patterns were mutually exclusive (Figure 6J and 6K).

At P10–P13, the mutant thymus consisted of numerous enlarged Hassall's corpuscle–like structures, made of arrays of K14- and K8-expressing keratinizing epithelial cells surrounding large keratin deposits (Figure 6L and 6O). There were numerous neutrophils and macrophages infiltrating the thymus in response to these keratins; hence, these structures could be called pyogranuloma. Varying degrees of differentiation-specific markers depending on the age of mice, in this case K1 and involucrin that are normally present only in Hassall's corpuscles, were also detected in mutant thymi (Figure S3). In these mice no thymocytes were detectable. BrdU incorporation was only observed in very few keratinizing epithelial cells, looking somewhat similar to the pattern of mature skin (Figure 6M). The diffuse expression of β-catenin was also present in these epithelial cells, and at this age fewer cells were positive for nuclear β-catenin staining (Figure 6N). As in the younger mutant mice, however, nuclear localization of β-catenin was only observed in K14-negative cells that looked like undifferentiated basal cells. In older P17 mice, the histopathology and keratin expression pattern of the mutant thymus was similar to that of P13 except for the fact that β-catenin expression became increasingly diffuse and appeared to colocalize with K8/K14 expression (data not shown). This coincided with fewer immature cells in the older mutant thymus.

Collectively, these results suggest that loss of *Apc* and consequent stabilization of β-catenin in K14-expressing TECs lead to their aberrant proliferation and differentiation to keratinocytes, causing massive squamous metaplasia, rather than to form either medullary or cortical TECs. Loss of proper TEC compartments consequently resulted in loss of thymocytes for maturation and the mice to be “athymic.”

## Discussion

*Apc* is implicated in the Wnt signaling pathway that is involved both in development and tumorigenesis. Human germline mutations in *APC* cause FAP [4,5], which is characterized by hundreds of adenomatous colorectal polyps, with an almost inevitable progression to colorectal cancer in the third and fourth decades of life. The phenotypical features of FAP and its variant, Gardner's syndrome, can be very variable. As well as colorectal polyps, these individuals can develop extracolonic symptoms, among which are upper gastrointestinal tract polyps, congenital hypertrophy of the retinal pigment epithelium, desmoid tumors, disorders of the maxillary and skeletal bones, and dental abnormalities [6]. While the heterozygous knockout mice for *Apc* develop adenomatous polyps predominantly in small intestine, the homozygous embryos die before gastrulation. To gain more insights into the effects of *Apc* loss in tissues other than gastrointestinal tract during life of animals and to circumvent the embryonic lethality associated with *Apc* nullizygosity, we created a mouse strain carrying a conditional allele of *Apc (Apc^CKO^)* in which exon 14 of the *Apc* is flanked by *loxP* sequences. The homozygous mice for the conditional allele are viable and indistinguishable to the normal mice, allowing us to study the roles of Apc in a tissue- and temporal-specific manner.

To assess the phenotypic consequences of inactivation of the *Apc* gene in cells that express K14, we created mice that are homozygous for the *Apc^CKO^* allele and contain a *K14-cre* transgene. These mice failed to thrive and died before weaning. They also exhibited hair, tooth, and thymus phenotypes.

### *Apc* and Hair Follicle Morphogenesis

Current models of hair follicle development suggest that the establishment of a regular array of placodes in the surface epithelium in response to the first dermal message is achieved through the competing activities of molecules that promote or repress placode fate [24]. There is accumulating evidence that activation of the Wnt signaling pathway in the dermis may be involved in establishing the first dermal signal. Experimental activation of epithelial β-catenin signaling (by expression of N-terminal–truncated, constitutively stabilized forms of β-catenin or ectopic expression of *Lef1*) induces ectopic follicles in both mouse and chick skin [25–27]. Conversely, down-regulation of β-catenin signaling (through *Lef1* knock-out, ectopic expression of Wnt inhibitor Dkk1 or conditional deletion of β-catenin in epidermis) results in loss of vibrissae and some pelage follicles in mice [28–30]. Our finding that *K14*-driven *Apc* loss in embryonic ectoderm resulted in irregularly spaced and often tightly clustered abnormal hair placode initiation and follicle morphogenesis (Figures 4F, 5B, 5D, and 5F) as well as in the development of multiple tooth buds (Figure 4O and 4P), is in line with the effects seen in activation of epithelial β-catenin signaling during placode formation, indicating the role of Apc in specification of embryonic ectodermal stem cells to produce a hair follicle. Given the role of Apc in down-regulation of β-catenin, loss of Apc would inevitably lead to altered expression of β-catenin in ectodermal cells during the hair placode formation, giving rise to aberrant follicular growth throughout the embryonic epidermis, including the footpads, where normally hairless (Figure 5B, 5D, 5F, and 5H). Of interest are the phenotypic similarities and differences between our KA mutant mice and the previously described K14-ΔN87βcat transgenic mice expressing stable β-catenin under the control of *K14* promoter [25] and K14-*Lef1* transgenic mice [27]. Surprisingly, our mutant mice shared more similarities with the K14-*Lef1* mice than the K14-ΔN87βcat transgenic mice. For example, the KA mutant mice displayed the disoriented and short, curly whiskers seen in K14-*Lef1*but not in K14-ΔN87βcat transgenic mice. In addition, KA mutant mice displayed a disorganized hair coat beginning with the emergence of neonatal hairs, similar to that observed in K14-*Lef1* mice, whereas in K14-ΔN87βcat mice, embryonic hair follicle morphogenesis was unaffected and other skin changes only emerged postnatally. The reason for this difference could be attributed to one or all of three possible contributing factors. (1) Although the K14 promoter itself is known to initiate the expression at E9.5 and elevated dramatically by E13.5–E14.5 [19,31], many factors could influence the expression of promoter/transgene construct in transgenic mice. One of the most important considerations has to do with the integration site of the transgene in the mouse genome. Depending on the location of integration, the transcriptional activity of even the same transgene could vary considerably. It is possible that the apparent lack of ΔN87βcat function in embryogenesis could be due to the delayed or weak expression of the K14-ΔN87βcat transgene compared to the intrinsic promoter. In contrast, Cre-mediated recombination is seen in E13.5 skin of *K14-cre* transgenic mice used in this study [16] and *K14-cre*–mediated truncation of the *Apc* gene is likely to have occurred by then, in time for the second wave of hair follicle morphgenesis. (2) Another important fact is that in the aforementioned transgenic model the overexpression of transgene is confined to the basal cells of the epidermis and the ORS of hair follicles, where the *K14* promoter is active. The expression of transgene is hence transient and stops once K14-expressing cells terminally differentiate. In contrast, in our model, *K14-cre*–mediated deletion of *Apc* would result in *Apc* mutation not only in *K14* promoter active cells, but remain so in all cell layers that derived from the *K14* promoter active progenitors. These derivatives include the suprabasal epidermis and the entire epithelium of the hair follicle. Stabilization of β-catenin in a broader population of cells could account for some of the phenotypic differences. However, we did not detect any elevated β-catenin expression in either K1- or K6-positive differentiated cells in postnatal mutant skin. This indicates that despite the absence of Apc and potential stabilization of β-catenin in derivatives of K14-expressing progenitors, elevated β-catenin expression, and subsequent cell fate determination may only take place in basal cells. (3) We cannot exclude the possibility that given the multifunctional properties of Apc, disruption of its functions other than down-regulation of β-catenin may also have contributed to the observed overt phenotype.

While some features of our mutant mice were similar to these transgenic mice, other phenotypic aspects were largely distinct. In addition to aberrant hair follicle morphogenesis, *K14*-driven loss of *Apc* caused formation of multiple tooth buds that, like hair follicles, were known to develop through inductive interactions between the epithelium and mesenchyme. This observation was similar to the ectopic tooth buds found in animals misexpressing Lef1 [27], but more severe and was also present at birth, indicating the effect of *Apc* loss during the initiation of embryonic tooth development, which was evident by aberrant *Shh* expression in E15.5 embryonic oral epithelium (unpublished data). It should be noted that although multiple tooth buds were histologically visible (Figure 4O and 4P), these teeth never broke out and the KA mutant mice appeared toothless. This unusual severe tooth defect is unique to these mutant mice. In addition, neither of the two transgenic mice was postnatally lethal as in the KA mutant mice. We did not find any obvious histopathological abnormalities in the internal organs of KA mice that could contribute to the lethality. However, the fact that all the mutants had lower weight (Figure 2F) with hardly any evidence of solid food in their stomach indicates that the mutants might have died of starvation. Dermal fat was reduced in the mutant skin, possibly as a consequence of poor nutrition caused by the absence of teeth. Since the weight loss in KA mutants started from P8–P10 while pups were still nursed by their mothers, starvation due to lack of teeth cannot be the sole cause of death, but is likely to be a contributing factor. Absence of teeth and mammary glands have been observed in mice deficient in Lef1 and ectopically expressing Dkk1 [29,30] but their absence was due to the block in development before the bud stage. Hence, neither loss nor excess of tooth bud formation allows proper development of teeth for mice to have a healthy diet and normal life. Mechanistic studies to understand how increased levels of β-catenin leads to altered skin and tooth phenotypes are under way.

### *Apc* and Thymus Organogenesis

In this study, we observed that *K14*-driven loss of *Apc* resulted in a small thymus with severe squamous metaplasia leading to the formation of numerous pyogranuloma and loss of proper meshwork structure for thymocyte maturation, rendering the mice athymic. Previous studies have shown that in normal adult thymus K14 expression is found together with K5 in the stellate medullary TECs, but not in association with K5^+^ TECs in the cortex or at the cortico-medullary junction. In addition, it has been demonstrated that K14^+^ TECs do not coexpress K8; hence, there are two distinct medullary subsets, namely a K8^−^K14^+^ stellate subset and a K8^+^K14^−^ globular subset [32]. In agreement with the previous results, in P3 normal thymus K14 expression was restricted to stellate medullary TECs (Figure 6D), whereas K8 expression was found throughout the TECs (unpublished data). We could not clarify whether these two keratins were coexpressed in the same TECs without double-staining. There were individual differences among mutants and these were prominently reflected in the histological abnormalities of the thymus at P3, but as the older surviving mutants all showed the same histopathologies of the thymus, the mutant thymi eventually seem to result in the same fate. It is unclear when *K14-cre* induction actually takes place in the mutant thymus, but as the population of cells showing a strong nuclear β-catenin staining as well as the cells expressing K14 were small and thymic epithelial compartments still existed in a mild P3 mutant thymus (Figure 6E and 6F), it seems that initial differentiation to medullary and cortical TECs and thymocyte colonization have already taken place prior to the major effects of *K14*-driven *Apc* loss. However, cells with nuclear β-catenin and K8^+^K14^+^ double-positive epithelial cells increased subsequently, associated with active proliferation in the latter group of cells and loss of TEC compartments. With age, β-catenin expression pattern became more diffuse and fewer epithelial cells showed nuclear localization but K8^+^K14^+^ cells remained, forming concentric structures of epithelial cells filled with hard keratin deposits and infiltrated with vast number of neutrophils and macrophages. Only a few epithelial cells were dividing and hardly any thymocytes were present by this time. These results suggest that with the deletion of *Apc* in TECs, stabililization and nuclear localization of β-catenin took place and subsequently these cells differentiated into keratinocytes that expressed both K14 and K8, similar to the basal cells of the skin, instead of TEC subsets. The expansion and differentiation of these keratinocytes lead to loss of proper thymic epithelial compartments and in turn produced and deposited the hard keratins that consequently caused vast amount of neutrophils and macrophages to infiltrate the thymus. These aberrant epithelial structures eventually have overtaken the whole of the thymus and driven out the colonized thymocytes. *K14*-driven loss of *Apc* and subsequent constitutive expression of β-catenin in TECs have therefore misdirected them to wrong epithelial cell fate, not allowing proper differentiation to either cortical or medullary TECs, which is essential for normal thymocyte development. This is not only evident from the lack of dividing thymocytes in the mutant thymus by P13, but also by the differential expression pattern of keratins, which were more skin-like than TEC-like. The importance of Apc function in thymic development has been demonstrated by thymocyte-specific loss of *Apc* by crossing a different strain of *Apc* conditional mice and LckCre transgenic mice [33]. Here, by *K14*-driven TEC-specific loss of *Apc,* we have demonstrated its importance in thymus development not only in thymocytes but also in TECs.

It is of interest that dental abnormalities, such as supernumerary and impacted teeth similar to those observed in our mutant mice, are frequently seen in patients with Gardner's syndrome, carriers of *APC* germline mutation [6]. The importance of odonto-stomatological examinations should hence be pointed out as a means of reaching a presumptive diagnosis, whose confirmation is vital to the patient. Further characterization of the mechanism of such developmental defects using our mouse model should provide important insights into *Apc* function in multiple organ systems and to give better insights into potential adverse events in human subjects.

In conclusion, we have shown that loss of *Apc* in *K14*-expressing embryonic cells causes aberrant morphogenesis in various skin appendages, including hair follicles and teeth, and abnormal thymus organogensis. Our results provide genetic evidence that expression of *Apc* is essential for regulation of proper cell fates in these organs that require epithelial–mesenchymal interactions.

## Materials and Methods

### Construction of targeting vectors.

To target the *Apc* locus, we obtained BAC clone RP23-233F17 that contains all the sequence of *Apc* except for the 5′ UTR and exon 1. Using a recombineering-based method for generating conditional mutations [12,13], subcloning of BAC and further modifications were conducted. The genomic sequence encompassing *Apc* exons 11 to 15 was first subcloned into pBR322 vector, and then a *loxP* site was introduced into intron 13 followed by *loxP*-*FRT*-PGK*neo^r^*-*FRT* selection cassette into intron14 of the *Apc* gene (Figure 1A).

### Generation of *Apc^CKO^* ES cells and mice.

The linearized targeting vectors were electroporated into 129/S-derived ES cells, WW6 cells [15]. All candidate G418-resistant ES clones were screened by long-range gene-specific PCR using Expand Long Template PCR System (Roche, Indianapolis, Indiana, United States), followed by sequencing to validate the correct insertion of the single *loxP* site and the selection cassette. Two ES clones with correct *Apc^CKON/+^* modification were injected into C57BL/6J blastocysts, after which chimeric mice with high levels of ES cell contribution were backcrossed to C57BL/6J females to produce heterozygous F1 offspring. The genomic DNA samples obtained from the tail of F1 offspring were subsequently analyzed by Southern blot analysis to further confirm the correct homologous recombination. By crossing the heterozygous mice to FLPe deleter mice [34], PGK*neo^r^* cassette was deleted in the germline by FRT-mediated recombination to generate mice with the final *Apc^CKO^* allele. *Apc^CKO^* heterozygous mice were crossed together to generate homozygous mice, and the homozygous offspring were interbred for maintenance.

### Generation of *Apc^Δ580^* mice.

*Apc^CKO^* heterozygote mice were crossed with Cre deleter mice, *EIIA-cre* transgenic mice that express Cre in early embryo [35], to knockout the *Apc* allele in the germline, consequently creating a knockout strain (Figure 1A). The resultant *Apc^Δ580/+^* mice were maintained by backcrossing to C57BL/6J females.

### Generation of *K14-cre; Apc^CKO/CKO^* mice.

The mice analyzed in this study were generated by crossing *Apc^CKO^* heterozygote mice of the F1 generation (C57BL/6J × 129/S background) with *K14-cre* transgenic mice (FVB background). *K14-cre; Apc^CKO/+^* male mice thus generated were then crossed with *Apc^CKO/CKO^* females to generate homozygous and heterozygous *Apc^CKO^* offspring either with or without *K14-cre*. The mice were intercrossed thereafter for maintenance.

### Genotyping of mice.

Genomic DNA from tips of mouse tails obtained at ~10 d of age was genotyped in a single PCR reaction with the following primers: Apc-Int13F2 (GAGAAACCCTGTCTCGAAAAAA) and Apc-Int13R2 (AGTGCTGTTTCTATGAGTCAAC), resulting in 320-bp and 430-bp products from the wild-type and conditional *Apc^CKO^* allele, respectively. For the detection of Cre-mediated deleted *Apc* allele, *Apc^Δ580^*, the primer Apc-Int14R4 (TTGGCAGACTGTGTATATAAGC) in combination with Apc-Int13F2 resulted in 500-bp product. Primers Cre-F1 (TCCAATTTACTGACCGTACACC) and Cre-R1 (CCGACGATGAAGCATGTTTAG) were used for detection of *cre* transgene in the germline, resulting in 300-bp products. Amplification was performed in a 25-μl volume containing 15 mM Tris-HCl (pH 8.3), 50 mM KCl, 2.5 mM MgCl_2_, 0.2 mM dNTP, 0.2μM of each primer, and 1.25 U of AmpliTaq Gold (Applied Biosystems, Foster City, California, United States). The reactions were heated for 10 min at 94 °C to heat-activate the enzyme followed by 30 PCR cycles at 94 °C for 30 s, 58 °C for 30 s, and 72 °C for 45 s, followed by the final extension for 5 min at 72 °C.

### Skin permeability assay.

Unfixed and freshly isolated E18.5 embryos were rinsed in PBS and then submerged in X-gal reaction mix at pH 4.5 (100 mM NaPO_4_, 1.3 mM MgCl_2_, 3 mM K_3_Fe(CN)_6_, 3 mM K_4_Fe(CN)_6_, and 1 mg/ml X-gal) at room temperature overnight [36]. At this pH in the absence of epidermal barrier, the solution penetrates epidermis and an endogenous β-galactosidase–like activity catalyzes production of a blue precipitate. After staining, embryos were washed twice in PBS, fixed overnight at 4 °C in 4% paraformaldehyde, and were photographed using a 35 mm Nikon digital camera.

### Skeletal preparations.

The skinned and eviscerated bodies of the oldest surviving KA mutants (P16–P17) and their age-matched littermates were placed into 1% potassium hydroxide (KOH) for 5 d. The bodies were transferred into a fresh solution of 1% KOH containing a few drops of 0.5% alizarin red S (Sigma, St. Louis, Missouri, United States) and left for another 5 d. The stained bodies were stored in glycerin and viewed under a dissection microscope.

### Analysis for tissue-specific recombination.

Genotype/tissue-specific recombination of the conditional allele was examined by both PCR and RT-PCR. DNA samples extracted from various tissues collected at the time of autopsy were examined by genotyping PCR as described. RNA extracted from various tissue homogenates in Trizol reagent were examined for expression of wild-type and truncated *Apc* alleles by SuperScript One-Step RT-PCR with Platinum Taq (Invitrogen, Carlsbad, California, United States), following manufacturer's protocol. Approximately 200 ng of total RNA from either skin, liver, or thymus from each genotype was reverse-transcribed using primers Apc-F546 (TGAGGAATTTGTCTTGGCGAG) and Apc-R721 (GCACTTCCCATGGCAATCATT), resulting in 528-bp and 313-bp products from the wild-type/*Apc^CKO^* alleles and *Apc^Δ580^* allele, respectively.

### BrdU labeling.

Mice were injected intraperitoneally with approximately 50 μg/g body weight of BrdU (Sigma) dissolved in PBS 2 h before their death. Tissue samples were fixed in Bouin's and processed as described below.

### Histological and immunochemical analysis.

Mutant mice and age-matching littermates were humanely killed at various ages by CO_2_ inhalation. Mice were skinned and pieces of skin were either snap-frozen in liquid nitrogen or immediately homogenized in Trizol reagent and stored at −80 °C until molecular analysis, or fixed flat on a piece of paper towel in Bouin's solution for histological and immunohistochemical examinations. The mice were then dissected for gross examination, various tissues were similarly collected for future molecular analyses, and then the whole body was fixed in Bouin's solution. The samples were then submitted to Rodent Histopathological Core for processing and histopathological examinations.

For immunohistochemistry, 5-μm paraffin-embedded tissue sections were deparafinized in xylene, followed by alcohol rehydration. After quenching endogenous peroxidases in 3% H_2_O_2_ in methanol, the slides were rinsed in distilled water, and an antigen retrieval step was carried out in a microwave oven for a total of 10 min in preheated citrate buffer (pH 6.0). The slides were then incubated with primary antibodies at room temperature overnight. Antibodies used were β-catenin (BD Transduction Lab, San Diego, California, United States), keratins 1, 5, 6, 14, involucrin, loricrin (Covance, Berkeley, California, United States), keratin 8 (Abcam, Cambridge, United Kingdom), Ki67 (Vector Laboratories, Burlingame, California, United States) and BrdU (Roche). Biotinylated secondary antibodies (donkey anti-rabbit and goat anti-mouse IgG, 1:250; Jackson ImmunoResearch, West Grove, Pennsylvania, United States), followed by the Vectastain Elite ABC kit (Vector Laboratories) were used for detection. The slides were stained with DAB and counterstained with Mayer's hematoxylin.

### In situ hybridization.

Section ISH using rat *Shh* probe [37] and whole-mount ISH using β-catenin probe [38] were performed as previously described [39,40]. Riboprobes labeled with DIG were detected with BM purple AP substrate precipitation solution (Roche).

## Supporting Information

Figure S1 Multiple Intestinal Neoplasia in *Apc^Δ580^* Mice (A) A representative appearance of small intestine of 4-month-old mouse. Scale bar, 1.5 mm.
(B) Histological analysis of longitudinal section of small intestine shown in (A) by H&E staining. Scale bar, 100 μm.
(C) The typical histopathological appearance of an adenoma shown in (B). Scale bar, 20μm.
(621 KB PPT)

Figure S2 Skin Permeability Assay and Neonatal Mice (A) Skin permeability assay of E18.5 embryos. Both normal (N) and KA mutant (M) embryos can exclude the blue dye at this age, showing no defects in skin barrier function. Note slight pigmentations in pinnnae and “birthmark” in the forehead of mutant embryo (B), which are present at P1 (C), and become prominent as they grow.
(749 KB PPT)

Figure S3 Immunochemical Examination of P13 Normal and Mutant Thymi (A–E) P13 normal thymus. (F–K) P13 mutant thymus. Stained with K8 (A), (D), (F), (I), K1 (B), (E), (G), (J), and involucrin (C), (H), (K). Scale bars, 100 μm for (A–C), (F–H), (J–K) and 20 μm for (D–E), (I). Note the lack of involucrin staining in normal thymus but varying degree of positive cells in mutant thymus.
(6.4 MB PPT)

### Accession Numbers

The GenBank ((http://www.ncbi.nlm.nih.gov/Genbank) accession numbers for the genes and gene products discussed in this paper are *Apc* (NM_007462), β-catenin (NM_007614), AXIN 2 (NM_004655), plakoglobin (NM_002230), Asef (NM_015320), kinesin superfamily–associated protein 3 (NM_014970), EB1 (NM_012325), human homolog of *Drosophila* Discs large (U13897), Lef1 (NM_010703), Dkk1 (NM_010051), K1 (NM_008473), K5 (NM_027011), K6 (NM_008476), K8 (NM_031170), K14 (NM_016958), involucrin (NM_008412), loricrin (NM_008508), and Shh (NM_009170).

## Abbreviations

APC: adenomatous polyposis coli
CKO: conditional knockout
E: embryonic day
ES: embryonic stem
FAP: familial adenomatous polyposis
FRT: FLP recognition target
ISH: in situ hybridization
K14: keratin 14
ORS: outer root sheath
P: postnatal day
TEC: thymic epithelial cell
